# CRISPR-mediated genomic repair of ARSA mutations in metachromatic leukodystrophy: a transformative step toward precision neuromodulation

**DOI:** 10.1097/MS9.0000000000004130

**Published:** 2025-10-28

**Authors:** Fakhar E. Mahin, Mahrosh Rubab, Mahnoor Fatima, Muhammad Talha, Ubaid Ur Rehman

**Affiliations:** a Department of Medicine, Fatima Jinnah Medical University, Lahore, Pakistan; b Department of Medicine, Rashid Latif Khan Medical University, Lahore, Pakistan; c Department of Medicine, Shaheed Ziaur Rahman Medical College, Rajshahi Medical University, Bogura, Bangladesh; d Department of Medicine, King Edward Medical University, Lahore, Pakistan

*To the Editor,*

Metachromatic leukodystrophy (MLD), an autosomal recessive lysosomal storage disease, is a life-threatening demyelinating disorder of the central and peripheral nervous systems, caused by a genetic mutation in the ARSA and PSAP genes, leading to a deficiency of the enzyme arylsulfatase A (ASA) and sphingolipid activator protein B. It is characterized by progressive motor and cognitive impairment with disease severity described by the presence of residual ASA enzyme activity. It is classified into three forms: late infantile, juvenile, and adult form^[1]^. This is in line with the TITAN Guidelines on the need for transparency in AI use in healthcare^[2]^. Current therapeutic strategies include bone marrow transplantation therapy, hematopoietic stem cell therapy, mesenchymal stem cell therapy, enzyme replacement therapy, and advanced gene therapy, each providing limited benefits with no definitive cure to date^[3,4]^.

The CRISPR/Cas system (Clustered Regularly Interspaced Short Palindromic Repeat/CRISPR-associated proteins) is an acquired immune system of bacteria and archaea, currently used as a genome editing tool for genetic and nongenetic diseases.^[5]^ In mammalian cells, it modulates the translation of desired genetic sequences through the nonhomologous end joining system and homology-directed repair techniques in all the phases of the eukaryotic cell cycle. This is performed by the acquisition of exogenous DNA with a specific Cas-mediated protospacer-adjacent motif sequence, transcription of specific CRISPR RNA, and multiplex editing of the target loci. This unique mechanism of action can restore the sulfatide activity of ASA by providing stable delivery of the functional enzyme, a novel therapeutic approach in MLD.^[3,5]^

Recent studies suggest that the synergism of a mutation-agnostic hematopoietic stem and progenitor cell gene therapy using CRISPR-Cas9 and AAV6 repair template has shown significant insertions and deletions (>87%) at the genomic site, leading to (>47%) gene integration. This led to a 30-fold rise in corrected ASA enzyme translation and expression, leading to effective treatment of MLD in the human models^[6]^. Genetically engineered neural models in mice showed enhanced ASA expression by crossing the blood–brain barrier (BBB) (*P* < 0.05) and reduced sulfatide storage in the brain and cerebellum (*P* < 0.05), making it an effective treatment modality for MLD^[4,7]^.

Despite advancements, there are many preclinical and clinical challenges to consider. It is a highly efficient yet costly therapy. The lack of long-term genomic studies, preclinical (newborn) screening, and early diagnosis, limited evidence of CRISPR’s efficacy, ability to cross the BBB, genomic graft rejections, defective genetic integration, and biomarkers to trace the ASA enzyme activity and disease progression make it difficult to assess the clinical translation of CRISPR in affected MLD individuals. Several ethical concerns exist in introducing this approach in children, particularly.

The novel advancement in MLD treatment with the CRISPR/Cas system brings a transformative genomic shift by restoring the enzyme activity in the affected neural and glial cells, reversing the neuropathology and the disease progression. This sheds light on a more coordinated approach by the scientific researchers across the globe to study the translational changes by CRISPR in a detailed approach. Through rigorous preclinical and clinical testing, a collaborative approach among researchers, stakeholders, policymakers, and affected MLD families, CRISPR brings a regime shift for the affected individuals and families^[3,4,6]^.

To conclude, CRISPR-mediated correction of the ARSA mutation provides a target-specific treatment modality for MLD. This scientific revolution requires a collaborative, multidisciplinary approach towards testing this therapeutic tool through ethical considerations, preclinical validation of off-target effects, and long-term clinical studies to investigate the durability and efficacy of CRISPR in neuromodulation for MLD.

## Data Availability

Not applicable to this study.

## Research registration unique identifying number (UIN)

No human subjects were involved.

## References

[1] Shaimardanova AA Chulpanova DS Solovyeva VV. Metachromatic leukodystrophy: diagnosis, modeling, and treatment approaches. Front Med (Lausanne) 2020;7:576221. 33195324 10.3389/fmed.2020.576221 PMC7606900

[2] Agha RA Mathew G Rashid R. Transparency in the reporting of artificial intelligence – The TITAN guideline. Prem J Sci 2025;10:100082.

[3] van Rappard DF Boelens JJ Wolf NI. Metachromatic leukodystrophy: disease spectrum and approaches for treatment. Best Pract Res Clin Endocrinol Metab 2015;29:261–73. 25987178 10.1016/j.beem.2014.10.001

[4] Sharma G Sharma AR Bhattacharya M. CRISPR-Cas9: A preclinical and clinical perspective for the treatment of human diseases. Mol Ther 2021;29:571–86. 33238136 10.1016/j.ymthe.2020.09.028 PMC7854284

[5] Makarova KS Haft DH Barrangou R. Evolution and classification of the CRISPR-Cas systems. Nat Rev Microbiol 2011;9:467–77. 21552286 10.1038/nrmicro2577 PMC3380444

[6] Antony JS Daniel-Moreno A Lamsfus-Calle A. A mutation-agnostic hematopoietic stem cell gene therapy for metachromatic leukodystrophy. CRISPR J 2022;5:66–79. 34882002 10.1089/crispr.2021.0075

[7] Audouard E Weber TJ Lerouge K. Successful treatment of adult MLD model mice by intravenous gene delivery of ssAAV9/ARSA. Mol Ther 2016;24:2097–107.

